# Myeloid-IL4Rα is an indispensable link in IL-33-ILCs-IL-13-IL4Rα axis of eosinophil recruitment in murine lungs

**DOI:** 10.1038/s41598-021-94843-9

**Published:** 2021-07-29

**Authors:** Sonika Patial, Brandon W. Lewis, Thao Vo, Ishita Choudhary, Kshitiz Paudel, Yun Mao, Dhruthi Singamsetty, Frank Brombacher, Yogesh Saini

**Affiliations:** 1 Department of Comparative Biomedical Sciences, School of Veterinary Medicine, Louisiana State University, 1909 Skip Bertman Drive, Baton Rouge, LA 70803 USA; 2 International Center for Genetic Engineering and Biotechnology, Cape Town Component & University of Cape Town, Health Science Faculty, IDM, Cape Town, South Africa

**Keywords:** Cytokines, Innate immune cells, Mucosal immunology

## Abstract

Increased eosinophil recruitment is a hallmark feature of eosinophilic disorders. Here, we delineated the key molecular and cellular players involved in physiological eosinophilic recruitment during normal postnatal lung development in mice. Physiological eosinophilic recruitment was consistently present in 7-, 10-, and 15-day-old neonatal mice, but not in 42-day-old mice. This feature was completely abolished in interleukin 33 (IL-33)-, interleukin 2 receptor gamma chain (IL2rγ)-, and interleukin 4 receptor alpha (IL4Rα)-knockout mice, but not in recombination activating gene 1 (Rag1)-knockout mice demonstrating an indispensable role for IL-33, innate lymphoid cells (ILCs), and IL4Rα in eosinophil recruitment. Interestingly, myeloid-specific IL4Rα-deficient (mye-IL4Rα^−/−^) mice had significantly reduced eosinophilia in the airspaces that was associated with reduced levels of IL-4 and IL-5 in the bronchoalveolar lavage fluid (BALF). Further, we tested the effect of myeloid-specific IL4Rα deficiency on IL-13-induced eosinophil recruitment into adult lung airspaces. Eosinophil recruitment into the airspaces was elevated in IL-13-treated WT mice but not in IL-13-treated mye-IL4Rα^−/−^ mice. Consistent with the degree of eosinophilia, the BALF levels of eosinophil recruitment-associated cytokines were significantly elevated in IL-13-treated WT but not in IL-13-treated mye-IL4Rα^−/−^ mice. These data establish that myeloid-IL4Rα is an indispensable component of the IL-33-ILCsIL-13-IL4Rα axis of eosinophil recruitment.

## Introduction

Eosinophil recruitment is a hallmark feature of multiple diseases including allergic asthma, parasitic infections, and food allergy^1,2^. Eosinophil recruitment contributes to disease-specific pathologies, such as airway remodeling in eosinophilic lung disorders by releasing a variety of cytokines and through interactions with other cell types, including epithelial and mast cells^1,3^. Accordingly, therapeutic strategies targeting eosinophil recruitment have been devised that aim at neutralizing these molecular and cellular components^4,5^. However, the specific sequence in which various molecular and cellular players function and the cell-specific roles of the molecular players involved in eosinophilic recruitment still remains unclear.

A number of molecular and cellular players have been shown to be involved in eosinophil recruitment. These include mediators, particularly interleukin 5 (IL-5), eotaxin, and interleukin 33 (IL-33), and cells including classical dendritic cells (cDC), innate lymphoid type 2 (ILC2) cells, and T-helper type 2 (*Th2*) cells^6–9^. Recently, employing a mouse model of chronic bronchitis, i.e., *Scnn1b*-Tg mice, we demonstrated that IL-33^10^ and IL2rγ^11^ are essential for the recruitment of eosinophils but adaptive immune response is dispensable in this process. Ablation of IL4Rα in *Scnn1b*-Tg mice also completely abolished eosinophil recruitment to the lung airspaces^12^. Spontaneous physiological airspace eosinophil recruitment has been reported during neonatal stages^12^, which likely either plays a role in lung development or reflects the maturation of the immune system. However, the roles of the aforementioned cellular and molecular players in physiological airspace eosinophil recruitment remain unclear.

In the present study, we tested the roles of four critical players, i.e., IL-33, IL2rγ (innate lymphoid immunity), Rag1 (adaptive immunity), and IL4Rα (a master regulator of *Th2* responses) in lung development-associated eosinophil recruitment. Most importantly, we tested the hypothesis that the myeloid cell-specific IL4Rα is essential for eosinophil recruitment. Towards this, we employed a novel strategy to achieve a near-complete deficiency of IL4Rα in myeloid cells and showed that achieving a near-complete deficiency of a gene may be critical in order to specifically delineate the role of myeloid cells in physiological and pathological conditions of the lung. Finally, we also tested the effect of myeloid-specific IL4Rα deficiency on IL-13-induced eosinophil recruitment into adult lung airspaces.

## Materials and methods

### Animals husbandry

All animal use procedures were approved by the Institutional Animal Care and Use Committee (IACUC) of the Louisiana State University. All methods were carried out in accordance with the IACUC guidelines and regulations. The study was carried out in compliance with ARRIVE guidelines. All the mice employed in this study were on C57BL/6 congenic background. Wild-type (WT; C57BL/6), Lysozyme M-Cre (LysMCre), and knockout (Rag1^−/−^, IL2rγ^−/−^, and IL4Rα^−/−^) mice were procured from Jackson laboratories. The IL-33^−/−^ mice were provided by Dr. Susumu Nakae (University of Tokyo, Japan)^13^. The floxed IL4Rα mice were provided by Dr. Frank Brombacher^14^. Both, IL4Rα^−/−^ and floxedIL4Rα, strains on BALB/c background were crossed back to the C57BL/6 background for more than 12 generations. We crossed LysMcre^+/+^ mice with IL4Rα^fx/fx^ to generate LysMcre monoallelic (LysMcre^+/−^/IL4Rα^fx/fx^) and LysMcre biallelic (LysMcre^+/+^/IL4Rα^fx/fx^, i.e., *mye*-IL4Rα^−/−^) mice. All the animals were identified using PCR genotyping. All mice used in this study were maintained in hot-washed, individually-ventilated cages on a 12 h dark/light cycle. Mice were fed a regular diet and water *ad libitum*. All the experiments were performed during the light period of 12 h dark/light cycle.

### BALF, blood, and bone marrow harvesting and analyses

Mice were lavaged as previously described^15^. BALF was centrifuged at 300xg for 5 min. The cell pellet was suspended in fresh phosphate-buffered saline (PBS) for total and differential cell counting as previously reported^16^. The cell-free BALF supernatant was saved at −80 °C for cytokine analyses. Mice were humanely euthanized and bone marrows were harvested from one femur per mouse for eosinophil counts. Peripheral blood was harvested from the posterior vena cava and eosinophil counts were performed in 150 µl of blood volume.

### Cytokine estimation

Cell-free supernatant was analyzed for the quantification of various soluble mediators as previously described^16^. Briefly, BALF was centrifuged at 300×*g* for 5 min and the cell-free supernatant was analyzed for cytokine levels. The BALF levels of IL-5, eotaxin, and IL-4 were determined using a Luminex-XMAP-based assay as per the manufacturer’s instructions (Millipore, Burlington, MA). Eosinophilia-relevant cytokines, i.e., CCL3 (MIP1α), CCL4 (MIP1β), CCL5 (Rantes), CCL7, CCL11 (Eotaxin 1), CCL12, CCL17, CCL22, and CCL24 (Eotaxin 2) were assessed in the BALF, as per manufacturer’s instructions (BioPlex Pro Mouse Chemokine panel, Hercules, CA). IL-33 was assessed in the BALF, as per the manufacturer’s instructions (LEGENDplex assay, BioLegend, San Diego, CA).

### IL-5/eotaxin- or IL-13-mediated lung eosinophilia

WT and *mye*-IL4Rα^−/−^ mice were intravenously injected with a cocktail of 0.5 µg IL-5 (PeproTech, Rocky Hill, NJ) and 1 µg CCL11/Eotaxin-1 (PeproTech, Rocky Hill, NJ). After a wait of 30 min, a cocktail of 1 µg IL-5 and 3 µg CCL24/Eotaxin-2 (PeproTech, Rocky Hill, NJ) was oropharyngeally delivered. At 2.5-h post-eosinophil chemoattractant treatment, mice were lavaged and analyzed for eosinophil counts. To induce IL-13-mediated lung eosinophilia, 5 µg of recombinant mouse IL-13 (R&D Systems, Minneapolis, MN) was oropharyngeally administered to each mouse on days 1, 3, 5, and 7. On day 8, mice were euthanized to collect BALF and determination of eosinophil counts.

### ILC2 flow cytometry

To induce IL-33-mediated ILC2 expansion, 1 µg of recombinant mouse IL-33 (R&D Systems, Minneapolis, MN) was oropharyngeally administered to each of the IL2rγ^−/−^, IL4Rα^−/−^, and *mye*-IL4Rα^−/−^ mice on days 1, 3, and 5. On day 6, mice were euthanized to harvest lung lobes in order to prepare single cell lung digest. Briefly, lungs were perfused by flushing 5 ml of PBS through the right ventricle of the heart. Perfused lungs were transferred to C-tubes (Miltenyi Biotec, Gladbach, Germany) containing 2.5 ml HBSS containing 500U/ml collagenase Type I (Worthington, Lakewood, NJ) and 200U/ml DNAase (Sigma Aldrich, St. Louis, MO). Lung tissues were digested using a GentleMACS dissociator (Miltenyi Biotec, Gladbach, Germany) followed by incubation on a rocker at 37 °C. Cell suspensions were filtered through 70 μm nylon cell strainer followed by centrifugation at 300×*g* for 10 min. Cells were resuspended in 10 ml of DMEM/F12 medium. Single -cell suspensions were stained with various fluorochrome-conjugated anti-mouse monoclonal antibodies for cellular phenotyping as follows: Lin (CD3e, B220, CD11b, TER-119, Gr-1, CD11c, FceR1α, CD8a, CD4), CD45, CD90.2, IL-33R, and CD278. Information for antibodies is included in Supplemental Table 1. Data were acquired using a CytoFLEX flow cytometer (Beckman Coulter Life Sciences, Indianapolis, IN) and analyzed using CytExpert software.

### Immunohistochemistry

Immunohistochemical staining for IL-33 was performed using procedure published previously^16–18^. Briefly, formalin-fixed, paraffin-embedded lung sections were deparaffinized with Citrisolv (2 × 5 min each) and were rehydrated with graded ethanol (100%, 95%, 70%, 30%, distilled water; 3 min each). Antigen retrieval was performed using a citrate buffer-based heat-induced method (heating slides in 10 mM sodium citrate solution [with 0.05% Tween 20; pH 6.0] at 95–100 °C for 30 min, followed by cooling to room temperature and quenching for 10 min with 3% hydrogen peroxide for blocking endogenous peroxidases. Sections were blocked with the blocking buffer for 20 min followed by primary antibody incubation with goat polyclonal to IL-33 (AB3626; R&D systems, Minneapolis, MN) at room temperature (RT) for 2 h. Sections were washed and incubated with diluted biotinylated secondary antibodies for 1 h at RT. The sections were then rinsed in deionized water (2 × 5 min each) and processed using VECTASTAIN Elite ABC HRP Kit (PK-6105, Vector Laboratories, Burlingame, CA), followed by chromogenic substrate conversion to insoluble colored precipitate using ImmPACT Nova RED HRP substrate Kit (SK-4800, Vector Laboratories, Burlingame, CA). Sections were counterstained with Gill’s Hematoxylin-I for 10 s, rinsed in deionized water, dehydrated with graded alcohol solutions, and coverslipped with VectaMount mounting media (H-5000, Vector Laboratories, Burlingame, CA). Immunostained slides were analyzed for the determination of positively stained cells. Briefly, photographs were captured under 40X objective of the ECLIPSE Ci-L microscope with DS-Fi2 camera attachment (Nikon, Melville, NY). Thereafter, captured images were processed using the ImageJ software (NIH) to determine the number of IL-33-stained cells and total number of hemotoxylin-stained nuclei^19^.

### Statistical analyses

One-way or two-way ANOVA followed by Tukey’s post hoc test for multiple comparisons was used to determine significant differences among groups. Significant differences between the two groups were determined by Student’s *t*-test assuming unequal variance. All data were expressed as mean ± standard error of mean (SEM). A *p*-value < 0.05 was considered statistically significant. Statistical analyses were performed using GraphPad Prism 8.0 (GraphPad Software, La Jolla, CA).

## Results

### Airspace eosinophilia is a consistent feature of neonatal lung development in mice

**Figure 1:**
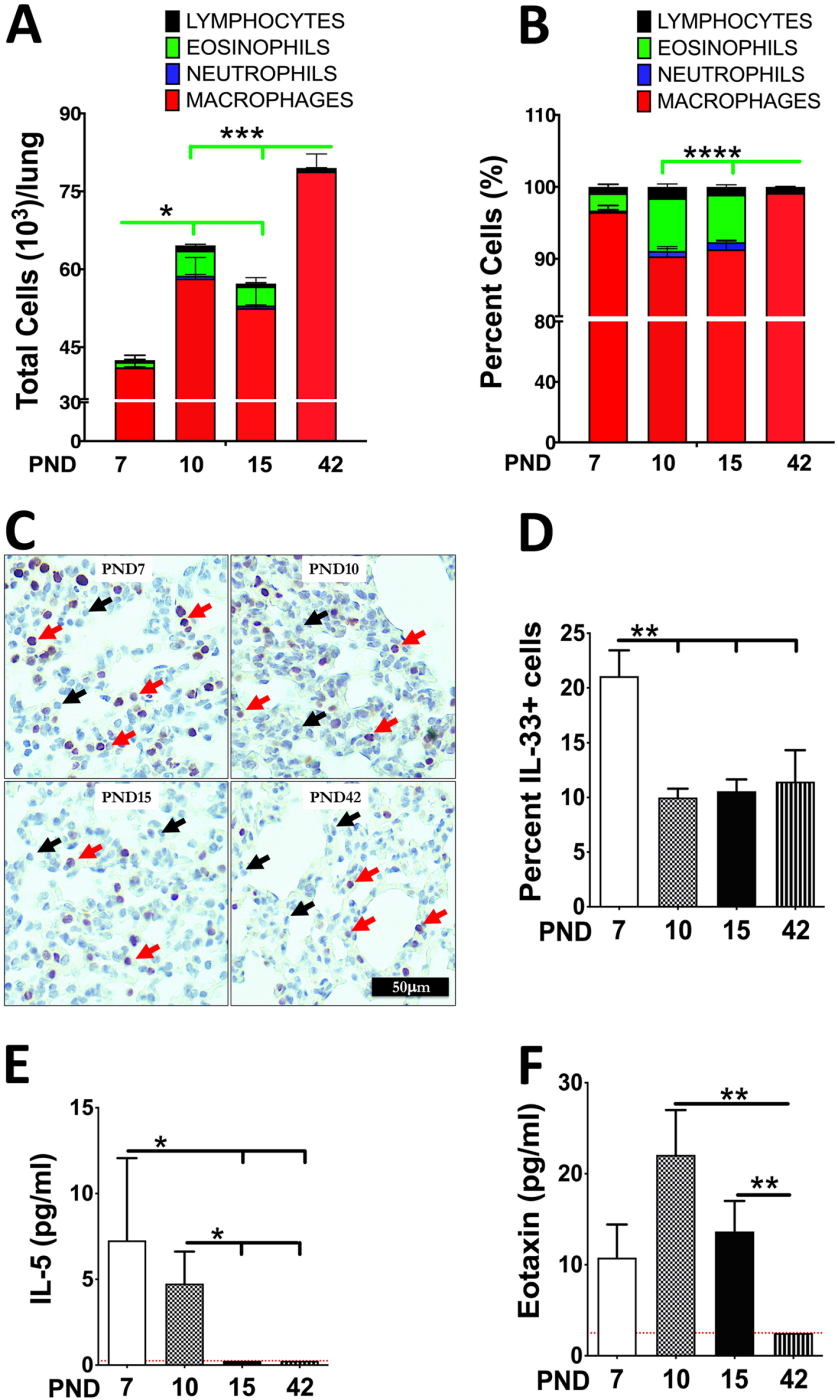
Airspace eosinophilia in the developing lungs of mice. Cell counts in BALF from mice at the age of postnatal day (PND) 7, 10, 15, and 42 (n = 5–10 per group). Differential cell counts (**A**) and relative percentages of constituent cell types (**B**) are shown as stacked bar graphs [macrophages (red), neutrophils (blue), eosinophils (green), and lymphocytes (black)]. Representative images of lung sections that were immunohistochemically stained for IL-33 (Red arrows; IL-33-stained cells, Black arrows; unstained cells) (**C**). Image J quantification of IL-33 immunostained sections (n = 5–6 per group) (**D**). Cytokine levels (pg/ml) of IL-5 (**E**) and Eotaxin (**F**) in cell-free BALF of mice at PND 7, 10, 15, and 42 (n = 5 per group). Error bars represent SEM. *p < 0.05, **p < 0.01, ***p < 0.001, ****p < 0.0001 using ANOVA followed by Tukey’s multiple comparison post hoc test. The lower limits of detection (indicated by red dotted lines) for IL-5 and Eotaxin were 0.25 pg/ml and 2.51 pg/ml, respectively. The results represent cumulative data on mice generated in two to three different litters.

Bronchoalveolar lavage fluid (BALF) eosinophilia has been previously reported in 10-day-old mice^12^. In order to access temporal pattern of eosinophilic recruitment during postnatal lung development, we performed a time-course analysis of airspace eosinophilic recruitment in C57BL/6 mice at four different age-points, i.e., post-natal day (PND) 7, 10, 15, and 42. In agreement with our previous study^15^, BALF from neonates at PND 7 contained ~ 2% eosinophils (Fig. 1A,B). In comparison, BALF from neonates at PND 10 and PND 15 contained ~ 6–7% eosinophils (Fig. 1A,B). At PND 42, however, BALF eosinophilia was not evident. These data suggest that a wave of eosinophilic recruitment, which initiates at PND 7, increases gradually through PND 10–15, and wanes towards PND 42 (Fig. 1A,B).

Next, we performed immunohistochemical staining for IL-33 on the formalin-fixed lung sections from 7-, 10-, 15-, and 42-day-old C57BL/6 mice. Image J quantification revealed a significantly higher number of IL-33-expressing cells, primarily alveolar type 2 cells, in 7-day-old neonates versus all other age points (Fig. 1C,D). Further, we determined the levels of IL-5, a cytokine essential for eosinophil survival and differentiation^20,21^, in the BALF of C57BL/6 mice. Consistent with elevated eosinophil counts, the BALF levels of IL-5 were elevated in the airspaces of neonates at PND 7 and 10 (Fig. 1E). The IL-5 levels were below the level of detection at PND 15 and PND 42. The levels of eotaxin, a potent eosinophil chemoattractant, were also elevated in 10-day-old C57BL/6 mice (Fig. 1F). These data suggest that the recruitment of eosinophils into the airspaces of 10/15-day-old mice is associated with IL-33 induction and IL-5 secretion at PND 7.

### The innate lymphoid system, but not the adaptive immune system, is essential for developmental eosinophilic recruitment

**Figure 2:**
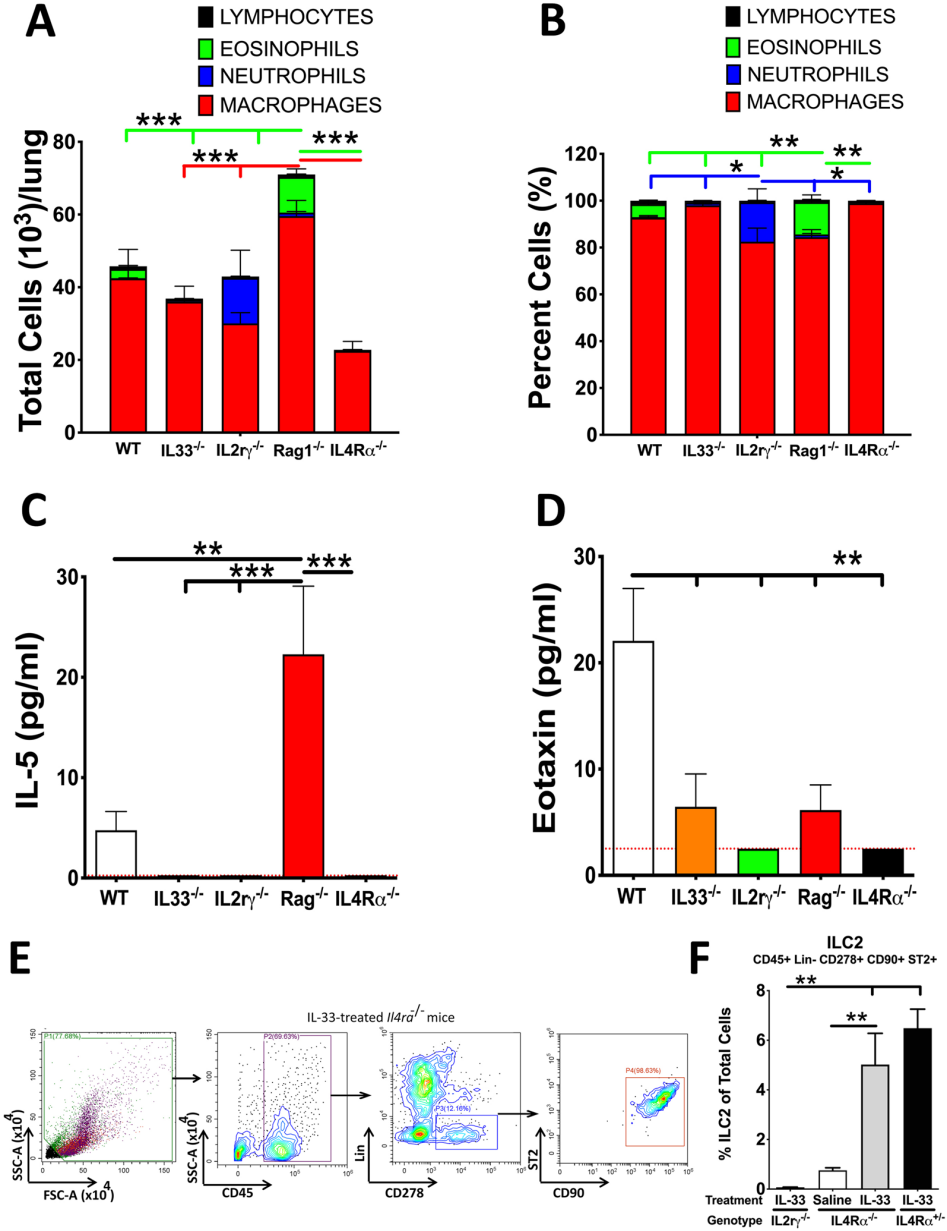
Airspace eosinophilia is regulated by the innate lymphoid system. Cell counts in BALF from 10-day-old wildtype (WT), IL-33^−/−^, IL2rγ^−/−^, Rag1^−/−^, and IL4Rα^−/−^ neonates (n = 5–14 per group). Differential cell counts (**A**) and relative percentages of constituent cell types (**B**) are shown as stacked bar graphs [macrophages (red), neutrophils (blue), eosinophils (green), and lymphocytes (black)]. Cytokine levels (pg/ml) of IL-5 **(C)** and Eotaxin (**D**) in cell-free BALF from 10-day-old wildtype, IL-33^−/−^, IL2rγ^−/−^, Rag1^−/−^, and IL4Rα^−/−^ neonates (n = 5 per group). The lower limits of detection (indicated by red dotted lines) for IL-5 and Eotaxin were 0.25 pg/ml and 2.51 pg/ml, respectively. Error bars represent SEM. **p < 0.01, ***p < 0.001 using ANOVA followed by Tukey’s multiple comparison post hoc test. (**E**) Gating Strategy and representative scatter plots for flow cytometric characterization of ILC2’s (CD45^+^Lin^-^ CD278^+^ CD90.2^+^ ST2^+^) in whole-lung single-cell suspension from IL-33-treated IL4Rα^−/−^ mice (n = 3 per group). (**F**) Percentage of ILC2s in whole-lung single-cell suspension from IL-33-treated IL2rγ^−/−^, saline-treated IL4Rα^−/−^, IL-33-treated IL4Rα^−/−^, and IL-33-treated IL4Rα^+/-^ mice. Error bars represent SEM. **p < 0.01, using ANOVA followed by Tukey’s multiple comparison post hoc test. The results represent cumulative data from mice generated in three different litters.

To test the role of IL-33 in eosinophil recruitment during postnatal lung development, we estimated eosinophil counts in BALF from 10-day-old IL-33 knockout (IL-33^−/−^) mice. In contrast to 10-day-old C57BL/6 mice (Fig. 2A,B), eosinophil recruitment was completely abolished in 10-day-old IL-33^−/−^ mice (Fig. 2A,B). Along the similar lines, IL-5 levels were below the lower limit of detection and eotaxin levels were significantly reduced in 10-day-old IL33^−/−^ mice compared to 10-day-old WT mice (Fig. 2C,D). These data suggest that IL-33 plays an indispensable role in eosinophilic recruitment during postnatal lung development.

Type 2 ILCs (ILC2s), *Th2*-counterpart of the innate lymphoid system, are activated in the presence of IL-33 and are the known producers of *Th2* cytokines^22^. *Th2* environment, as determined by the presence of *Th2* cytokines, i.e., IL-4 and IL-13, is essential for eosinophil recruitment^7^. To verify the role of *Th*2 environment promoting ILC2s in developmental eosinophilic recruitment, we analyzed BALF from 10-day-old interleukin 2 receptor gamma chain (γc) knockout (IL2rγ^−/−^) mice for the presence of eosinophils and eosinophil chemoattractants. Similar to IL-33^−/−^ mice, the eosinophils (Fig. 2A,B), IL-5 (Fig. 2C), and eotaxin (Fig. 2D) were undetected in the BALF from IL2rγ^−/−^ mice.

In addition to the absence of ILCs and NK cells, the IL2rγ^−/−^ mice also lack adaptive immune cells^11,23^. To investigate whether the absence of the adaptive immune system in the IL2rγ^−/−^ mice caused the absence of airspace eosinophilia, we analyzed recombinase activation gene 1 knockout (Rag1^−/−^) mice that lack mature adaptive immune cells^24^. BALF eosinophil recruitment (Fig. 2A,B) as well as levels of eosinophil chemoattractants (Fig. 2C,D) in BALF were not compromised in these mice. In fact, Rag1^−/−^ mice exhibited exaggerated eosinophil recruitment (Fig. 2A,B) and higher concentration of IL-5 (Fig. 2C) as compared to C57BL/6 WT counterparts. These data suggest that the innate lymphoid system is essential for eosinophilic recruitment during postnatal lung development. Further, the data also indicate that the adaptive immune system may have a regulatory/suppressive effect on eosinophil recruitment.

### IL4Rα signaling is required by ILCs for developmental eosinophil recruitment

Since ILC2s are known to produce IL-5^25^, we anticipated that the eosinophil recruitment and IL-5 production will remain unaffected in the absence of IL4Rα. Therefore, we investigated whether IL4Rα is required for eosinophil recruitment. Interestingly, the IL4Rα^−/−^ neonates were completely devoid of BALF eosinophils (Fig. 2A,B) and eosinophil recruitment-associated cytokines (Fig. 2C,D). The role of IL4Rα in ILC2 function has been reported previously^26^. To determine whether the IL4Rα^−/−^ mice have intact ILC2s, we oropharyngeally challenged IL2rγ^−/−^, IL4Rα^−/−^, and IL4Rα^+/-^ mice with IL-33 and performed flow cytometry to analyze the proportions of ILC2s (CD45 + Lin-CD278 + CD90.2 + ST2 +) in the lungs of these mice strains (Fig. 2E, F, Supplemental Fig. 1). As expected, IL-33-treated IL2rγ^−/−^ mice were completely devoid of ILC2s. (Fig. 2F, Supplemental Fig. 1). IL-33 treatment, however, resulted in a significant increase in the ILC2 populations in IL4Rα^−/−^ as well as IL4Rα^+/-^ mice compared to saline-treated IL4Rα^−/−^ mice, in which only ~ 0.75% of analyzed cells were ILC2s (Fig. 2E, F, Supplemental Fig. 1). These data suggest that IL4Rα deletion does not compromise IL-33 induced expansion in ILC2 population in lungs and that ILC2 products, most likely IL-13 or IL-4, signal through IL4Rα, to result in the recruitment of eosinophils.

### IL4Rα ligands, i.e., IL-13 and IL-4, are required for eosinophil recruitment into the developing airspaces

IL-13, a prominent *Th2* cytokine, is a known product of ILC2 cells^27^. Our data strongly suggest that the IL-33-ILCs-IL4Rα axis is a key pathway of eosinophil recruitment (Fig. 2). Therefore, we next determined whether the secretory levels of IL-13 were different in C57BL/6, IL-33^−/−^, IL2rγ^−/−^, and IL4Rα^−/−^ neonatal PND 10 mice, i.e., (1) elevated in 10-day-old C57BL/6 neonates, (2) diminished in 10-day-old IL-33^−/−^ and IL2rγ^−/−^ neonates, and 3) elevated in 10-day-old IL4Rα^−/−^ neonates.

**Figure 3:**
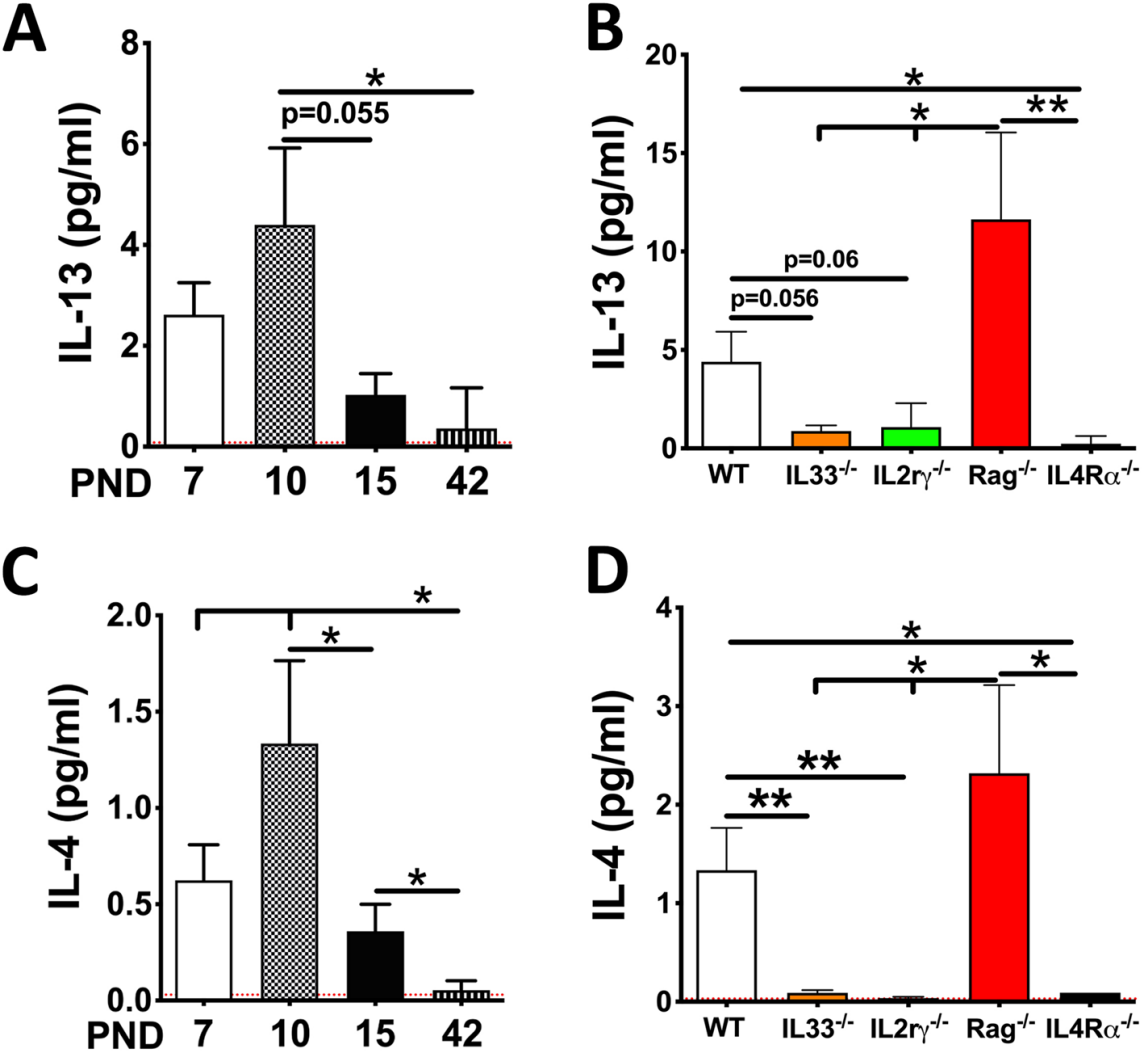
Levels of ligands for IL4Rα receptor in airspaces. (**A**) Cytokine levels (pg/ml) of IL-13 in BALF from WT mice at the age of postnatal day (PND) 7, 10, 15, and 42 (n = 5 per group). (**B**) Cytokine levels (pg/ml) of IL-13 in BALF from 10-day-old wildtype (WT), IL-33^−/−^, IL2rγ^−/−^, Rag1^−/−^, and IL4Rα^−/−^ neonates (n = 5 per group). **(C)** Cytokine levels (pg/ml) of IL-4 in BALF from WT mice at PND 7, 10, 15, and 42 (n = 5 per group). **(D)** Cytokine levels (pg/ml) of IL-4 in BALF from 10-day-old WT, IL-33^−/−^, IL2rγ^−/−^, Rag1^−/−^, and IL4Rα^−/−^ neonates (n = 5 per group). Error bars represent SEM. *p < 0.05, **p < 0.01, using ANOVA followed by Tukey’s multiple comparison post hoc test. The lower limit of detection (indicated by red dotted lines) for IL-4 and IL-13 were 0.03 pg/ml and 0.08 pg/ml, respectively. The results represent cumulative data from mice generated in three different litters.

Our analyses revealed that C57BL/6 neonates had significantly elevated levels of IL-13 at PND 10 (Fig. 3A). As expected, the IL-13 levels were significantly diminished in IL-33^−/−^ and IL2rγ^−/−^ neonates (Fig. 3B). While IL-13 levels were exaggerated in Rag1^−/−^ neonates, interestingly, the IL-13 levels were significantly diminished in IL4Rα^−/−^ neonates (Fig. 3B). These data suggest that the sufficiency of IL4Rα signaling is essential for increased levels of IL-13. In other words, both ILC2s as well as IL4Rα-bearing cells are required for IL-13 production and eosinophil recruitment. Along the similar  lines, BALF levels of IL-4 were elevated in 10-day-old C57BL/6 (Fig. 3C), diminished in IL-33^−/−^, IL2rγ^−/−^, and IL4Rα^−/−^ neonates, and exaggerated in Rag1^−/−^ neonates (Fig. 3D). Accordingly, we conceptualized that IL33-ILC2 interaction induces the production of low levels of IL-13 and/or IL-4, that is further amplified via IL-13-IL4Rα-mediated signaling in IL4Rα-expressing cells. Whether the ILC2-derived IL-13 binds back to the IL4Rα on ILC2s, in an autocrine manner, or to the IL4Rα on other non-ILCs, in a paracrine manner, is not known.

### Myeloid-IL4Rα signaling is essential for developmental eosinophil recruitment

We furthered our investigation on identifying a key IL4Rα-bearing cell type involved in the amplification of IL-13/IL-4 levels and, thus, eosinophil recruitment via increased production of IL-5. We speculated that a resident cell-type, e.g., myeloid cells [macrophages and classical dendritic cells (cDC)] or epithelial cells, would be a realistic candidate for this investigation. Therefore, we hypothesized that myeloid-IL4Rα responds to ILC2-derived IL-13 and recruits eosinophils via amplification of *Th2* molecular signals, including IL-13, IL-4, and IL-5. Towards this, first, we generated a *mye*-IL4Rα^−/−^ mouse strain through an innovative approach that involved increasing the copy number of LysMcre from monoallelic (used in most studies published to date) to biallelic, in order to accomplish efficient deletion of myeloid-IL4Rα. To ascertain that the recombination in floxed alleles is restricted to the myeloid population in the lung parenchyma, we first generated bitransgenic mice carrying biallelic LysMcre and Rosa-mTom^Fl/Fl^/mEGFP floxed reporter allele^15^. Fluorescent imaging of the lungs from these mice revealed that the recombination in floxed reporter allele was restricted to the myeloid compartment as indicated by the expression of mEGFP (Supplemental Fig. 2). Of note, all other parenchymal cells were mTom positive indicating that the biallelic LysMcre does not target the non-myeloid cell population (Supplemental Fig. 2). These mice were healthy and to date, we have not noticed any phenotypic abnormalities in these mice. This is not completely unexpected given the fact that IL4Rα^−/−^ whole-body knockout mice are also phenotypically normal under homeostatic conditions^28^.

**Figure 4:**
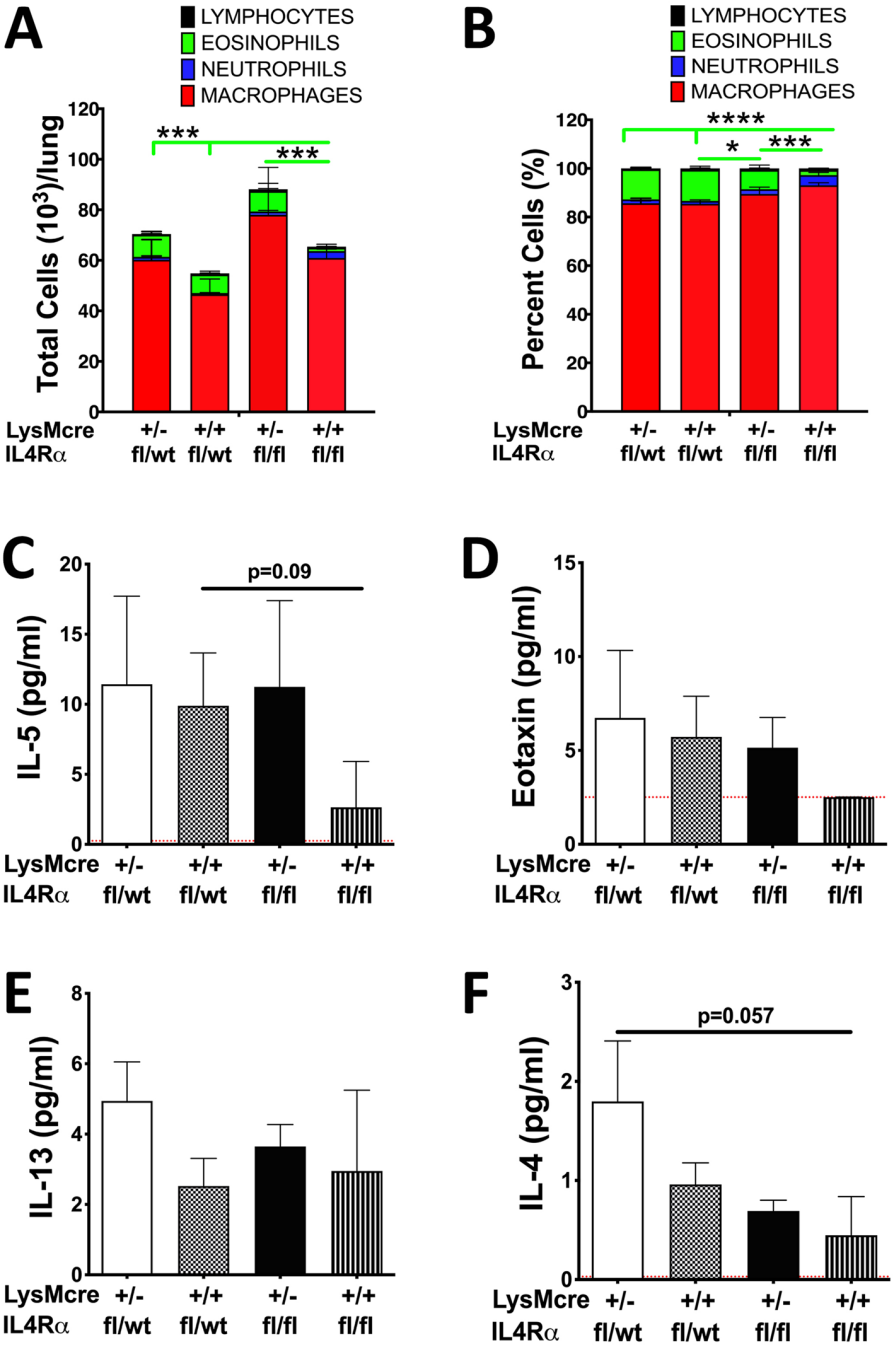
Eosinophilic recruitment into the lung airspaces is compromised in myeloid-specific IL4Rα-deficient mice. Cell counts in BALF from 10-day-old LysMcre^+/−^/IL4Rα^fl/wt^, LysMcre^+/+^/IL4Rα^fl/wt^, LysMcre^+/−^/IL4Rα^fl/fl^, and LysMcre^+/+^/IL4Rα^fl/fl^ neonates. Differential cell counts **(A)** and relative percentages of constituent cell types (**B**) are shown as stacked bar graph [macrophages (red), neutrophils (blue), eosinophils (green), and lymphocytes (black)]. Cytokine levels (pg/ml) of IL-5 **(C),** Eotaxin (**D**)**,** IL-13 (**E**)**,** and IL-4 (**F**) in cell-free BALF from 10-day-old LysMcre^+/-^/IL4Rα^fl/wt^, LysMcre^+/+^/IL4Rα^fl/wt^, LysMcre^+/−^/IL4Rα^fl/fl^, and LysMcre^+/+^/IL4Rα^fl/fl^ neonates. Error bars represent SEM. *p < 0.05, ***p < 0.001, ****p < 0.0001, using ANOVA followed by Tukey’s multiple comparison post hoc test. n = 4 per group. The lower limit of detection (indicated by red dotted lines) for IL-4, IL-13, IL-5, and Eotaxin were 0.03 pg/ml, 0.08 pg/ml, 0.25 pg/ml, and 2.51 pg/ml, respectively. The results represent cumulative data from mice generated in five different litters.

The percent eosinophils recovered were comparable between LysMcre^+/−^/IL4Rα^fx/wt^ (~ 12%) and LysMcre^+/+^/IL4Rα^fx/wt^ (~ 12%) suggesting that the presence of biallelic cre and inactivation of one allele of IL4Rα does not result in a reduction in BALF eosinophilia (Fig. 4A,B). However, the percent eosinophils were reduced to ~ 8% in mice with monoallelic LysMcre (LysMcre^+/−^/IL4Rα^fx/fx^) (Fig. 4A,B). Interestingly, a significant reduction in BALF eosinophilia (~ 2%) was observed in LysMcre^+/+^/IL4Rα^fx/fx^ mice. Furthermore, IL-5 levels were reduced, although not significantly, in the BALF from LysMcre^+/+^/IL4Rα^fx/fx^ mice compared to all the other controls (Fig. 4C). The eotaxin was present above detection levels in BALF from all the three controls but below the detection limits in the BALF from LysMcre^+/+^/IL4Rα^fx/fx^ mice (Fig. 4D). While IL-13 levels were comparable between all the four groups (Fig. 4E), reduced levels of IL-4 were trending towards significance in LysMcre^+/+^/IL4Rα^fx/fx^ mice as compared to all the other controls (Fig. 4F). These data suggest that myeloid IL4Rα is essential for the maintenance of IL-4 levels in the airspaces, and eosinophil recruitment. We demonstrate that a nearcomplete depletion of myeloid IL4Rα using biallelic LysMcre allele is dramatically more effective as compared to monoallelic LysMcre allele in elucidating this effect (Fig. 4). These data, for the first time, establish an indispensable role of IL4Rα signaling in myeloid cells in the recruitment of eosinophils.

### Myeloid-IL4Rα signaling is essential for IL-13-induced lung eosinophilia

**Figure 5:**
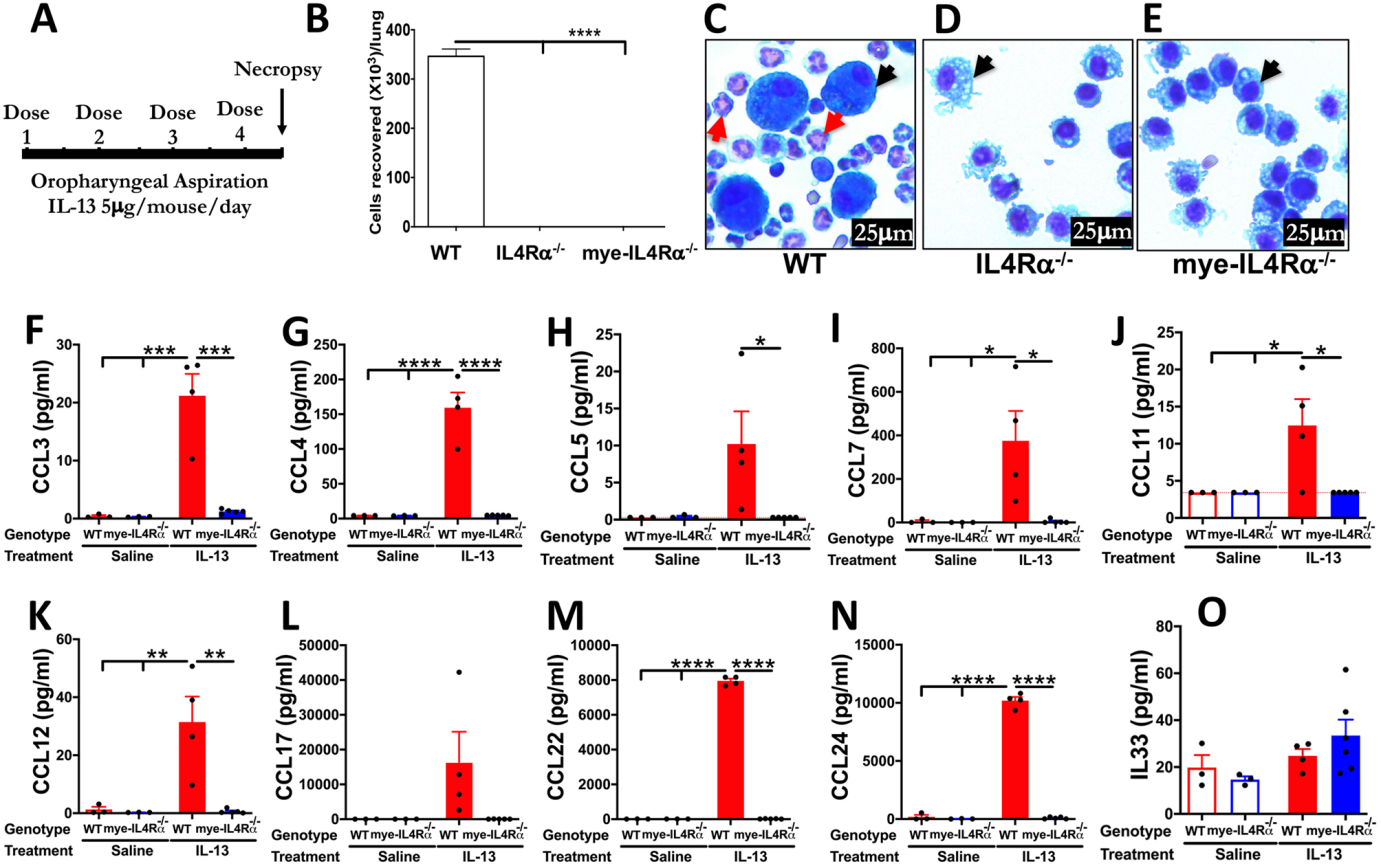
Myeloid-IL4Rα is essential for IL-13 and IL-5/eotaxin-induced lung eosinophilia and eosinophil chemoattractants are significantly reduced in the BALF of IL-13 treated mye-IL4Rα^−/−^ versus IL-13 treated WT mice. Scheme for IL-13-induced lung eosinophilia (**A**). Total number of eosinophils recovered from IL-13 treated WT, IL4Rα^−/−^ and mye-IL4Rα^−/−^ mice (n = 4 per group) (**B**). Representative photomicrograph of BALF cytospins from IL-13-treated WT (**C**), IL4Rα^−/−^ (**D**), and mye-IL4Rα^−/−^ (**E**) mice (red arrows, eosinophils; black arrows, macrophages). Error bars represent SEM. *p < 0.05, **p < 0.01, ***p < 0.001, ****p < 0.0001, using ANOVA followed by Tukey’s multiple comparison post hoc test. Cytokine levels (pg/ml) of CCL3 (MIP1α) (**F**), CCL4 (MIP1β) (**G**), CCL5 (Rantes) (**H**), CCL7 (**I**), CCL11 (Eotaxin 1) (**J**), CCL12 (**K**), CCL17 (**L**), CCL22 (**M**), CCL24 (Eotaxin 2) (**N**)**,** and IL-33 (**O**) in cell-free BALF from saline- or IL-13-treated WT and mye-IL4Rα^−/−^ adult mice. The lower limit of detection for CCL3 (MIP1α), CCL4 (MIP1β), CCL5 (Rantes), CCL7, CCL11 (Eotaxin 1), CCL12, CCL17, CCL22, and CCL24 (Eotaxin 2) were 0.23 pg/ml, 4.0 pg/ml, 4.0 pg/ml, 0.27 pg/ml, 0.42 pg/ml, 3.41 pg/ml, 0.22 pg/ml, 5.03 pg/ml, 0.42 pg/ml, 0.60 pg/ml, and 4.06 pg/ml, respectively. The lower limit of detection for CCL5 (Rantes) and CCL11 (Eotaxin 1) are indicated by red dotted lines. Error bars represent SEM. *p < 0.05, **p < 0.01, ***p < 0.001, ****p < 0.0001, using 2-way ANOVA followed by Tukey's multiple comparison post hoc test. n = 3–5. Results in panels B-E are representative of three independent experiments. Data in panels F-O were generated using BALF from 3 to 5 independent animals.

IL-13 is known to induce eosinophilic recruitment to the airspaces of mice^7^. To test whether IL-13-induced eosinophilic recruitment requires myeloid-specific IL4Rα expression, we oropharyngeally exposed WT, IL4Rα^−/−^ and *mye*-IL4Rα^−/−^ adult mice to recombinant mouse IL-13 (Fig. 5A). As expected, the IL-13-treated adult WT mice had elevated levels of eosinophils in the BALF (Fig. 5B,C). However, the IL-13-treated IL4Rα^−/−^ adult mice had no signs of eosinophilic recruitment (Fig. 5B,D). Similarly, the BALF from *mye*-IL4Rα^−/−^ adult mice was completely devoid of eosinophils (Fig. 5B,E). These data suggest that the myeloid-specific IL4Rα sufficiency is essential for IL-13-mediated recruitment of eosinophils.

To identify the levels of eosinophil chemokines in the BALF of IL-13-treated WT mice and to determine the effect of myeloid cell-specific deletion of IL4Rα on the BALF levels of eosinophil chemokines, we analyzed CCL3 (MIP1α), CCL4 (MIP1β), CCL5 (Rantes), CCL7, CCL11 (Eotaxin 1), CCL12, CCL17, CCL22, and CCL24 (Eotaxin 2). The BALF levels for all of these chemokines were comparable between saline-treated WT and *mye*-IL4Rα^−/−^ adult mice (Fig. 5F–N). Consistent with the increased eosinophilic infiltration in IL-13-treated WT mice, the BALF levels of CCL3 (MIP1a), CCL4 (MIP1b), CCL7, CCL11, CCL12, CCL22, and CCL24 (Eotaxin 2) were significantly elevated in IL-13-treated versus saline-treated WT mice (Fig. 5F–N). Consistent with the absence of eosinophilic infiltration in IL-13-treated *mye*-IL4Rα^−/−^ mice, all the eosinophil chemokines were comparable between IL-13-treated and saline-treated *mye*-IL4Rα^−/−^ mice (Fig. 5F–N). BALF IL-33 levels were comparable between the IL-13- as well as saline-treated WT and *mye*-IL4Rα^−/−^ mice (Fig. 5O).

**Figure 6:**
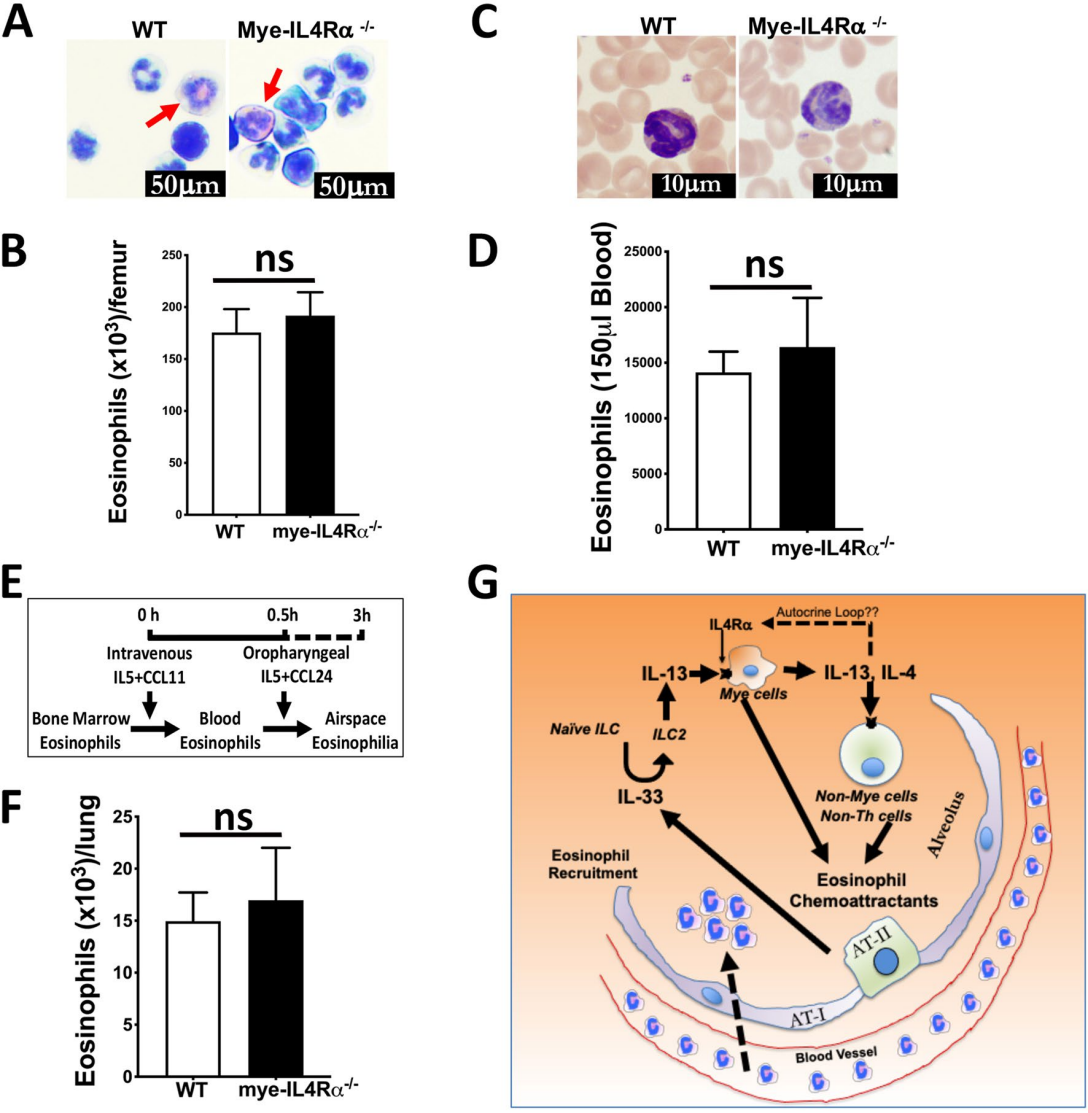
IL4Rα deletion in myeloid cells does not compromise responsiveness of eosinophils to IL-5/eotaxin-mediated recruitment into the airspaces. Total number of eosinophils recovered in the femoral bone marrow (**A**: bone marrow cytospins, red arrows depict eosinophils; **B**: eosinophil counts) and blood (**C**: blood cytospins; red arrows depict eosinophils; **D**: eosinophil counts) from WT and mye-IL4Rα^−/−^ mice (n = 4 per group). Scheme for IL-5/CCL11/CCL24-induced lung eosinophilia (**E**). Total number of eosinophils recovered from IL-5/CCL11/CCL24-treated WT and mye-IL4Rα^−/−^ mice (n = 4 per group) (**F**). Error bars represent SEM. Student’s t-test. ns; non-significant. (**G**) A conceptual overview of the cellular and molecular players during eosinophil recruitment. Release of IL-33 from the alveolar epithelial cells induces the development of ILC2s. ILC2-derived IL-13 binds to IL4Rα on myeloid cells and the latter produces detectable levels of IL-13 and IL-4. On the downstream, eosinophil chemoattractants are produced by myeloid cells, or indirectly by non-myeloid cells, that in turn, recruit eosinophils into the airspaces. Data in panels A-F were generated using BALF from 3 to 5 independent animals.

Lysozyme M is known to be expressed in eosinophils^29^, therefore, to ascertain that the potential deletion of IL4Rα in the eosinophils did not affect their abilities to populate the bone marrow and blood, we assessed eosinophil counts in the bone marrow and blood from WT and *mye*-IL4Rα^−/−^ adult mice. The number of eosinophils recovered in the bone marrow harvested from the femur (Fig. 6A,B) and blood (Fig. 6C,D) were comparable between WT and *mye*-IL4Rα^−/−^ mice. Finally, to ascertain that the deletion of IL4Rα in the eosinophils did not affect their abilities to respond to the chemoattractants, we experimentally induced IL-5/eotaxin-mediated lung eosinophilia in the WT and *mye*-IL4Rα^−/−^ adult mice (Fig. 6E). Both, WT and *mye*-IL4Rα^−/−^ adult mice had comparable eosinophil counts in the BALF after the IL-5/eotaxin treatment (Fig. 6F).

## Discussion

Eosinophil recruitment is a typical response associated with *Th2* inflammation^30^. Based on the current understanding of *Th2* immune responses, the release of master regulator cytokines, i.e., IL-33, IL-25, and TSLP, from stressed/injured epithelial cells is believed to initiate *Th2* immune responses in the airspaces^31^. These cytokines have been suggested to stimulate the differentiation of naïve innate lymphoid cells (ILC) into type 2 ILC (ILC2) that in turn produce *Th2* cytokines, IL-13 and arguably IL-4^32–34^. These cytokines act on various cell types via IL4Rα, that result in the production of IL-5 and other eosinophil chemoattractants^35^. Therapeutic strategies targeting eosinophil recruitment are under clinical trials for eosinophilic disorders including allergic asthma^4,5^. However, the responses to these therapeutic strategies are not very promising perhaps due to limited understanding of the players involved in the eosinophil recruitment. In this study, we dissected the eosinophil recruitment axis using a mouse model of development-associated lung eosinophilia.

The exact nature of stimulus causing eosinophil recruitment during lung development is unclear, however, we speculated that the active remodeling/apoptosis in the respiratory tract might cause leakage of intracellular IL-33 into the developing airspaces. While IL-33 was not detected in the BALF collected from C57BL/6 neonates at PND 10, the immunohistochemical staining of lung sections for IL-33 protein revealed a significantly increased percentage of IL-33-expressing cells at PND 7 versus PND 10, 15, and 42. These data revealed that the appearance of increased numbers of IL-33-expressing cells, most likely alveolar type II cells, coincides with the appearance of eosinophilic recruitment into the airspaces. Consistent with these findings, IL-33 deletion completely abolished eosinophil recruitment into the developing lungs sugesting that the recruitment of eosinophils into the airspaces of the developing lungs is an IL-33-mediated response. This also suggests that, in the absence of IL-33, the presence of IL-25 and TSLP is not adequate for the recruitment of eosinophils into the developing lung airspaces. This finding is consistent with our recent report where we demonstrated that IL-33 deficiency results in a complete abolition of eosinophil recruitment to the mucoobstructive airways of *Scnn1b*-Tg + mice^18^.

Since multiple cell types in the lungs harbor IL1RL1(ST2), an IL-33 receptor, we tested whether the eosinophil recruiting axis traverses through IL-33 → ILCs and/or IL-33 → non-ILCs. Therefore, we hypothesized that the IL-33 → ILCs axis is critical for eosinophil recruitment. Using IL2rγ^−/−^ mice that lack innate lymphoid cells, we clearly demonstrated that IL-33 indeed acts through ILCs to result in eosinophil recruitment. IL-33 is essential for the development of ILC2 subtypes^27,32^, however, since eosinophils were absent in both IL-33^−/−^ and IL2rγ^−/−^ mice, we suggest that only the ILC2s are required for the recruitment of eosinophils.

The Rag1^−/−^ mice, that possess all the non-amnestic equivalents of adaptive immune cells, i.e., ILCs, had no defect in the eosinophilic recruitment. Indeed, these mice exhibited significantly elevated eosinophil counts and proportions. Consistent with the increase in eosinophil counts, the Rag1^−/−^ mice had elevated levels of *Th2* cytokines including IL-13, IL-4, and IL-5. These data suggest that the adaptive immune cells suppress the production of IL-5 and thus eosinophilic infiltration. These observations are in line with our recent study where we have shown that the Rag1 deficiency in *Scnn1b*-Tg mice, a mouse model of mucoobstructive lung disease, results in exaggerated IL-5 production^11^. The cells of the adaptive immune system, including *Th* and regulatory T cells (Tregs), are known producers of IL-10, an immunosuppressive cytokine^36–38^. Furthermore, mast cell-derived IL-2 is known to promote Treg expansion and IL-10 production that, in turn, has been shown to inhibit ILC2-mediated eosinophilic recruitment^39^. Accordingly, we speculate that the immunosuppressive effects of IL-10 on cells that produce eosinophilic chemoattractants result in mitigated eosinophil recruitment under normal conditions, an effect that was lost in Rag1^−/−^ mice resulting in exaggerated eosinophilia.

IL-13 is a known product of ILC2s in various experimental models including pulmonary fibrosis^10,40^, helminth infection^32,41,^ and allergic asthma^25,42^. Consistent with these reports, IL-13 levels were lower in IL-33^−/−^ as well as IL2rγ^−/−^ mice in developing lungs (Fig. 3B). Interestingly, IL-13 levels also remained at baseline in IL4Rα^−/−^ mice despite the fact that they were IL-33 and ILCs sufficient (Fig. 3B). Consistent with IL-13 levels in five experimental groups (Fig. 3B), IL-4 levels were also present at the baseline levels in IL-33^−/−^, IL2rγ^−/−^, and IL4Rα^−/−^ mice (Fig. 3D). These data suggest that the IL4Rα, in addition to IL-33 and IL2rγ, are required for the increased production of IL4Rα ligands i.e., IL-13 and IL-4. IL-5 is another known product of ILC2s in various experimental models with eosinophilic inflammation including influenza virus infection^43^ and allergic asthma^25,42^. Consistent with IL-13 and IL-4 levels, IL-5 levels were also diminished in the BALF from IL-33^−/−^, IL2rγ^−/−^, and IL4Rα^−/−^ mice (Fig. 2C). The WT mice, on the other hand, had increased levels of IL-13 (Fig. 3B), IL-4 (Fig. 3D), and IL-5 (Fig. 2C), which correlated with the presence of airspace eosinophils (Fig. 2A,B) in these mice. Collectively, these data suggest that IL4Rα-mediated signaling is essential for the production of IL-13, IL-4, and IL-5.

To delineate the cellular players in the IL4Rα-signaling pathway, we further hypothesized that IL4Rα on one of the resident cell types, i.e., either epithelial or myeloid cells within the airspaces is essential for eosinophil recruitment. In this study, we focused on IL4Rα signaling in the myeloid cells. Since myeloid cell populations in the airspaces are developmentally heterogeneous, we reasoned that, in order to decipher the role of IL4Rα-myeloid signaling in eosinophil recruitment, it is critical to achieve a near-complete ablation of IL4Rα in myeloid cells at all stages of development. LysMCre-mediated recombination, although a very efficient system, does not induce a complete and efficient recombination^44^. We suggest two reasons for this inefficient recombination in the context of lungs: (1) the dosage of Cre recombinase from a single Cre allele may not be sufficient for an efficient recombination, and/or (2) since the cellular recruitment into the airspaces is a highly dynamic phenomenon, myeloid cells, particularly macrophages, are constitutively recruited into the airspaces, particularly, during the time of lung injury/insults. However, newly recruited myeloid cells may have limited or delayed LysM promoter activation and therefore may be resistant to efficient Cre-mediated recombination.

In order to test whether the dosage from a single Cre allele is insufficient, we compared myeloid deletion using a single allele (monoallelic) Cre recombinase with a biallelic Cre recombinase, to delete IL4Rα in myeloid cells. Interestingly, this simple strategy, i.e., increasing the dosage of Cre recombinase, made a dramatic impact. We found that myeloid-IL4Rα deletion using biallelic Cre completely abolished physiological eosinophil recruitment into the airspaces while monoallelic Cre resulted in only partial inhibition of eosinophil recruitment. Because Cre transgene is inserted into the endogenous Lysozyme M promoter through a targeted locus disruption approach, the biallelic LysMCre status renders the host, Lysozyme M deficient. Therefore, to circumvent the untoward effects of loss of Lysozyme M, we included a critically important biallelic LysMCre control (LysMcre^+/+^/IL4Rα^wt/fx^) mice in our experiments. The data from this control confirmed that the effects observed in myeloid-IL4Rα mice were not a result of loss of Lysozyme M or Cre-recombinase toxicity. The benefits of biallelic over monoallelic deletion, however, could be model-specific. For example, while the inactivation of IL4Rα in LysMcre^+/−^/IL4Rα^-/fx^ mice, a genotypic combination stoichiometrically equivalent to LysMcre^+/+^/IL4Rα^fx/fx^, conferred resistance against cryptococcus infection^45^, no protection in eosinophilic recruitment was reported in LysMcre^+/−^/IL4Rα^−/fx^ mice against allergic airway disease^46^.

Lysozyme M is known to express in eosinophils^29^, therefore, we reasoned that the potential deletion of IL4Rα in the eosinophils might affect their maturation, exit from the bone marrow, or their abilities to express chemokine receptors, i.e., eotaxin receptors^47^ (e.g., CCR2, 3, and 5) responsible for their recruitment to the lungs. However, our analyses of *mye*-IL4Rα^−/−^ adult mice did not reveal any defects in the eosinophil population within the bone marrow or the blood. Further, IL-5 and eotaxin administration was able to induce eosinophil recruitment into the airspaces of *mye*-IL4Rα^−/−^ adult mice. These data suggest that the myeloid-specific IL4Rα deletion within the eosinophils themselves does not compromise their differentiation into mature cells or their expression of eotaxin receptors, e.g., CCR2, 3, and 5.

Our knowledge of the cell-specific roles of IL4Rα sufficiency in eosinophilic recruitment is very limited. Accordingly, we tested whether IL4Rα deficiency in myeloid cells affects the eosinophil recruitment process. The absence of BALF eosinophilia in *mye*-IL4Rα^−/−^ mice and associated reduction in BALF levels of IL-4, IL-5, and eotaxin suggest that IL4Rα expression in myeloid cells is essential for eosinophil recruitment. Based on these findings, we propose that IL4Rα-bearing myeloid cells, act as sensors for low levels of ILC2-derived IL-13, that in turn, further amplifies the levels of IL-4 to perpetuate eosinophilic recruitment. Whether this feedforward mechanism exists in an autocrine or paracrine manner remains untested. One caveat of this study is that the LysMcre induces recombination in floxed alleles in multiple cell types of the myeloid lineage including macrophages, neutrophils, basophils, and classical dendritic cells^48,49^. Therefore, it is still not clear as to which of these myeloid cell populations is required for eosinophil recruitment. Therefore, additional studies employing macrophage-, neutrophil-, basophils-, and cDC-specific IL4Rα-deficient strains are warranted.

In conclusion, this study delineates (Fig. 6G) an eosinophil recruitment axis, IL-33-ILC2-myeloid-IL4Rα-IL-4/IL-13-eosinophil recruitment, and reveals several innovative findings including (1) IL-33 is essential for eosinophil recruitment, (2) innate lymphoid system is indispensable for eosinophil recruitment, (3) adaptive immune system is dispensable for eosinophil recruitment, and (4) myeloid-specific IL4Rα is essential for the recruitment of eosinophils and production of eosinophil chemoattractants. Collectively, our data show that IL4Rα-bearing myeloid cells are essential for eosinophil recruitment under physiological conditions. Importantly, we also provide a simple yet novel approach to achieve an effective myeloid deficiency of IL4Rα. More importantly, we believe that the results obtained, particularly in pulmonary research, using single allelic LysMcre-mediated recombination in other studies may need to be revisited. In summary, our findings suggest that targeting myeloid cell IL4Rα may be an effective, cell-specific strategy, in eosinophilic lung disease therapeutics.

## Supplementary Information

Supplementary Legends.

Supplementary Information 2.
