# Supplementary material for: Myeloid-IL4Rα is an indispensable link in IL-33-ILCs-IL-13-IL4Rα axis of eosinophil recruitment in murine lungs
Source: Sci Rep. 2021 Jul 29;11:15465. doi: 10.1038/s41598-021-94843-9 (PMC8322172; doi:10.1038/s41598-021-94843-9)
Supplement: Supplementary file 1 — Supplementary Legends. [file 41598_2021_94843_MOESM1_ESM.docx]

**Supplemental Figure 1:** Gating Strategy and representative scatter plots for flow cytometric characterization of ILC2’s (CD45^+^Lin^-^ CD278^+^ CD90.2^+^ ST2^+^) in whole lung single cell suspension from designated groups.

**Supplemental Figure 2:** Fluorescent photomicrograph showing biallelic LysMcre induced recombination in Rosa-mTom^Fl/Fl^-mEGFP floxed reporter allele. Arrows point to LysM-expressing myeloid cells.
