# Supplementary material for: Myeloid-IL4Rα is an indispensable link in IL-33-ILCs-IL-13-IL4Rα axis of eosinophil recruitment in murine lungs
Source: Sci Rep. 2021 Jul 29;11:15465. doi: 10.1038/s41598-021-94843-9 (PMC8322172; doi:10.1038/s41598-021-94843-9)
Supplement: Supplementary file 2 — Supplementary Information 2. [file 41598_2021_94843_MOESM2_ESM.pdf]

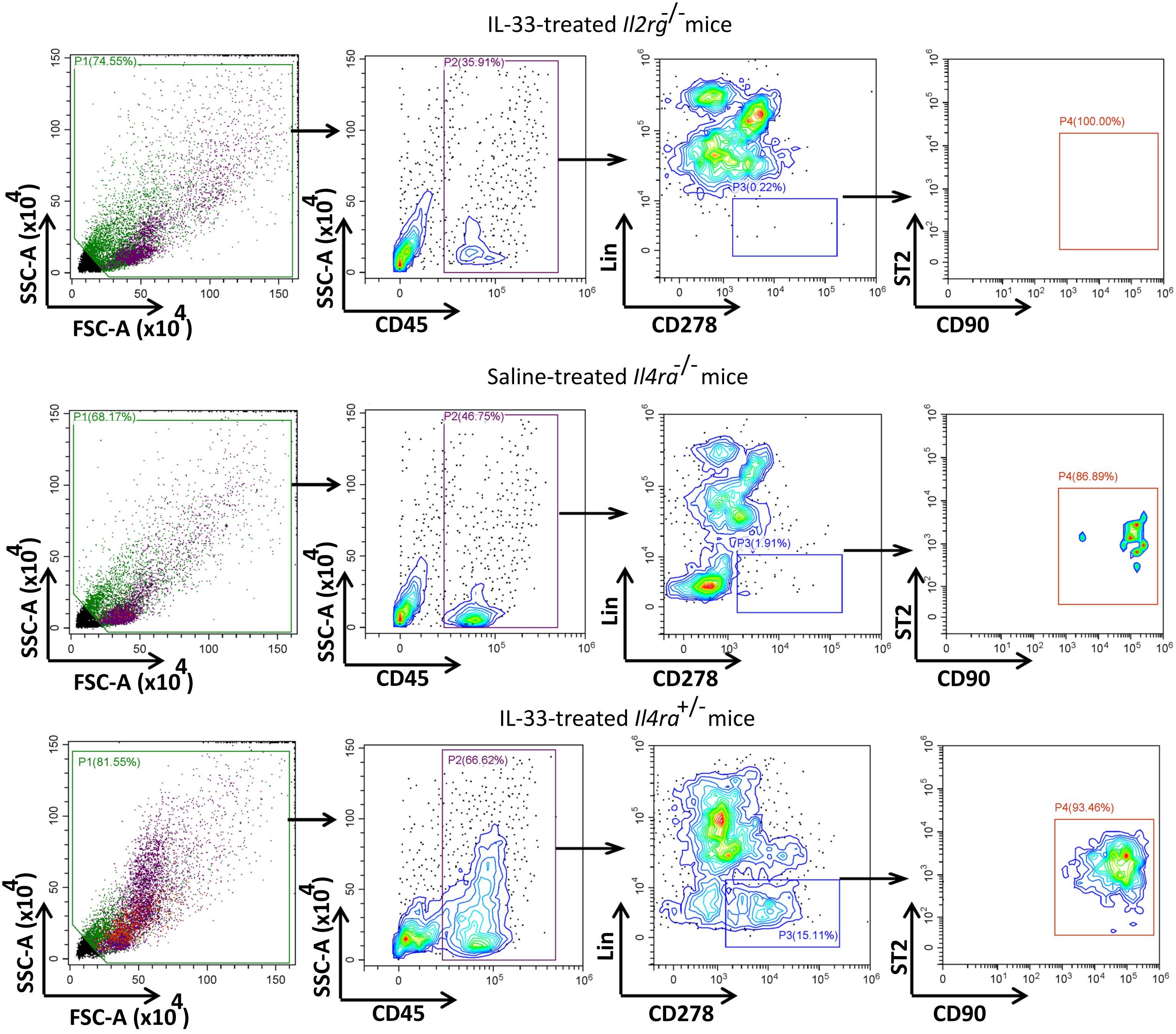

Supplemental Figure 1

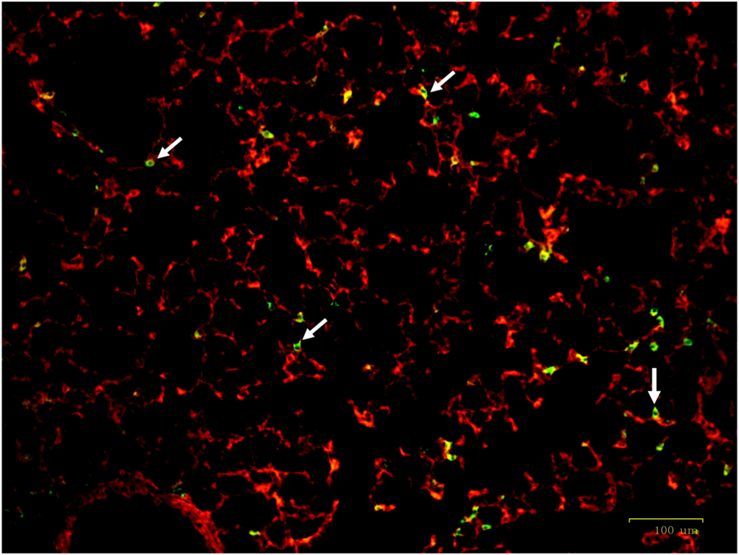

Supplemental Figure 2

Supplemental table 1: Flow cytometry antibody information

Antibody	Clone	Fluorophore	Company (Catalog number)	Concentration
CD3$^-$	17A2	Lineage cocktail-FITC (CD3, B220, CD11b, TER-119, Gr-1)	133302 (BioLegend)	20 ul/test
B220$^-$	RA3-6B2			
CD11b$^-$	M1/70			
TER-119$^-$	TER-119			
Gr-1$^-$	RB6-8C5			
CD11c$^-$	N418	FITC	117306 (BioLegend)	0.5 mg/ml
FCeR1α^-	MAR-1	FITC	11-5898-85 (Life Technology)	0.5 mg/ml
CD8a$^-$	53-6.7	FITC	100706 (BioLegend)	0.5 mg/ml
CD4$^-$	GK1.5	FITC	100406 (BioLegend)	0.5 mg/ml
CD45$^+$	30-F11	AF700	103128 (BioLegend)	0.2 mg/ml
IL-33R (ST2)$^+$	D1H9	BV421	145309 (BioLegend)	0.2 mg/ml
CD278 (ICOS)$^+$	C398.4A	APC	313510 (BioLegend)	0.2 mg/ml
CD90.2 (Thy-1.2)$^+$	53-2.1	Super Bright 600	63-0902-82 (Life Technology)	0.2 mg/ml
